# Malaria elimination without stigmatization: a note of caution about the use of terminology in elimination settings

**DOI:** 10.1186/1475-2875-13-377

**Published:** 2014-09-22

**Authors:** Catherine Smith, Maxine Whittaker

**Affiliations:** School of Population Health, University of Queensland, Herston, QLD Australia

**Keywords:** Stigma, Surveillance, Mobile populations, Malaria elimination

## Abstract

This commentary offers a note of caution about the negative social impact that may be inadvertently generated through malaria elimination activities. In particular, the commentary is concerned with the practice of describing people who remain at risk of malaria in low transmission settings as ‘hotpops’ or ‘reservoirs of infection’. The authors argue that since those at risk of malaria in elimination settings are often already socially marginalized – such as migrants, indigenous groups, ethnic minorities and poor rural communities – that care should be taken to avoid implementing programmes in ways that may inadvertently add to the social stigmatization of those most at risk of malaria in a low transmission setting. Programmes should avoid using language that identifies particular groups as a source of infection, and instead begin a broader shift in orientation toward engaging constructively with communities within elimination strategies. Programmes should promote monitoring and evaluation to ensure that unintended negative consequences such as stigma do not occur; advocate for appropriate resourcing (human, financial, other) to minimize the risk of short cuts being used to achieve an end game that may discriminate against specific groups; and strengthen community engagement activities in elimination setting to avoid targeting stigmatized groups and to empower communities to prevent outbreaks and re-introduction of malaria. In this way malaria elimination can be achieved without stigmatization.

## Background

As malaria incidence declines globally, malaria is becoming increasingly concentrated in particular localities and demographic groups [1]. In countries nearing elimination where malaria is now scarce, a growing consensus is concerned with addressing malaria amongst populations at high risk of malaria, who are often referred to as ‘hotpops’ (hot populations) or reservoirs of infection [1–3]. This commentary expresses concern about the possible adverse impacts that may be generated through the use of terminology that identifies groups as sources of infection, imported malaria and ongoing transmission. This is of particular significance in an elimination setting, since many of the populations that elimination programmes wish to target – such as migrant workers, displaced persons, indigenous peoples, ethnic minorities and remote communities – are often already stigmatized and socially disadvantaged in many ways.

While malaria is not stigmatized in itself, this commentary offers an early warning about the adverse social impacts that may result if programmes are implemented in a way that inadvertently legitimizes already held social prejudices against marginalized peoples who are also at risk of malaria. The authors propose four central points: (i) Malaria elimination is likely to increasingly involve marginalized people and, therefore, social and ethical considerations should be more strongly integrated into elimination strategies; (ii) Although malaria is not in itself stigmatized, there is the potential for stigma to arise within an elimination context if marginalized groups become seen as sources of ongoing infection and imported malaria; (iii) Language matters as it frames the perceived implications of disease; and (iv) There is a potential to achieve malaria elimination with minimal adverse social impact, if social perspectives are incorporated into programme strategies as more countries head towards elimination.

### Malaria elimination is likely to increasingly involve marginalized people, so social and ethical considerations should become integrated into elimination strategies

Many countries that are in an elimination or pre-elimination phase have observed a spatial shift of malaria into concentrated localities (hotspots) together with an epidemiological shift of malaria into more narrowly defined demographic groups [1–3]. The populations frequently mentioned in the recent malaria elimination literature include forestry workers [4, 5], agricultural workers [6–8], migrant workers [9–11], displaced persons [9, 12–16], ethnic minorities [17], and border communities [18–20], many of which lack adequate health services.

It is important for programmes to recognize that as malaria elimination advances, those who remain at risk of malaria are likely to be people who are already in some way socially disadvantaged, as is often the case for migrants, displaced persons, ethnic minorities and rural communities that remain poor when the broader society is experiencing economic growth. This exclusion may result from poverty, lack of citizenship, racism, exclusion from services, language barriers, class discrimination or other forms of social prejudice [12, 14, 16, 18]. These forms of structural violence can strongly affect people’s lives, shape the social determinants of health, and in some cases act as a barrier to programme success. This can be seen, for example, in situations where poverty, language differences or lack of citizenship act as a barrier to prevention and treatment services [12, 14, 16].

Many have argued for the importance of incorporating social perspectives into malaria control programmes to increase programme effectiveness and to minimize adverse social impacts [21–23]. The authors argue that in situations when malaria is scarce and more likely to affect groups that are already socially marginalized, that it is even more important for elimination programmes to become more aware of the shifting socio-political context of malaria transmission and to take measures to develop programmes in a way that minimizes adverse social impact and maximizes the benefits of elimination to communities. This should not be left until the final stages of elimination, but should become incorporated into elimination strategies now as more countries gear up towards elimination.

### Although malaria is not in itself stigmatized, there is the potential for stigma to arise within an elimination context if marginalized groups become seen as sources of ongoing infection

While malaria is not usually associated with stigma, malaria elimination programmes may generate stigma in situations where the disease is scarce and concentrated in pockets of people who are already marginalized. In order to understand the potential for malaria elimination programmes to generate stigma, it is important to recognize that stigma is not an attribute of a disease in itself but rather is a product of the way in which society constructs the perceived social, moral and political implications of a disease. Importantly, these social meanings change over time, as the social and political context of a disease shifts and as official and popular discourses surrounding disease change.

Classic sociological approaches see stigma as a process through which individuals become marginalized from broader society if they are seen as possessing an undesirable attribute that differentiates them from the perceived norm [24]. Stigma was historically associated with conditions that mark a person physically, such as leprosy, or diseases that were culturally interpreted through a strong moral lens, such as sexually transmitted infections and mental illness [24]. Stigma is often embedded in a claim that there has been a violation of “shared attitudes, beliefs and values” [25]: p50. Weiss sees that stigma develops from cultural meanings of illness and exaggerated fears that surround the illness [26]. These cultural meanings are embedded in the contemporary socio-political context of illness. However the cultural meanings of diseases change significantly over time, often reflecting contemporary social tensions about race, class and morality [27–29].

Fears surrounding stigmatized illnesses often reflect broader socio-political fears to a much greater extent than they reflect any rational concern about disease itself. For example, early 20th century understandings of hookworm in the US were deeply influenced by racist ideologies. Hookworm was seen as a ‘disease of laziness,’ and discourses surrounding hookworm described African Americans as a public health menace [29]. Another example can be found in the cholera outbreak in Venezuela in 1992 and 1993, where the threat of cholera became strongly associated with indigenous people and the urban poor, who were widely seen as uneducated and refusing to conform to modern society [27]. Both public health and media discourses surrounding the cholera outbreak became heavily tainted by racial and class discrimination, in particular leading indigenous peoples to be disproportionately blamed for the epidemic [27]. Although fear of contagion is often evident within stigma [25, 26] non-infectious diseases can also become stigmatized as other forms of social discrimination become attached to an illness. For example class discrimination can be projected onto smokers [30]; people with eating disorders can be stigmatized for breaching gender norms [31–33]; while people with chronic fatigue syndrome are often seen as failing to be productive citizens [34]. Stigma is generated as a dominant group suggests that an individual or minority group has somehow become an imagined threat to society.

The practice of labelling migrants, indigenous groups and poor rural communities as ‘hotpops’ or ‘reservoirs of infection’ is concerning, since these discourses are directed at groups that often already face multiple forms of structural violence. Although malaria may be culturally normalized in some societies where it is still common, malaria programmes operating in elimination settings should be aware that programmes are increasingly seeking to target narrowly defined demographic groups that are often already social vulnerable. As the authors argued in a review of the literature on malaria and population mobility [35], recent elimination programmes have painted an exaggerated image of mobile populations as segregated from local communities, engaged in illegal activities and avoiding authorities. Williams and colleagues [14] have expressed concern about the way that elimination discourse describes displaced persons as a threat to programme success, while comparatively little attention is given to their health needs. While the discussion about ‘reservoirs of infection’ is clearly intended to inform accurate surveillance strategies, it also uses highly negative language to describe groups as a source of infection. While not suggesting that malaria elimination programmes are currently generating negative impacts through elimination discourse, the potential for this to occur is evident. As malaria reduces it will be important for malaria elimination programmes to consider how malaria is understood within this changing socio-political context – especially when managing malaria imported malaria and disease outbreaks – and to develop strategies that are aware of the changing socio-political context in which elimination programmes operate.

### Language matters as it frames the perceived implications of disease

An important starting point for this shift in orientation will be a more considered use of language within malaria elimination. Although public health programmes are, of course, intended to protect public health, history shows many examples where public health discourse has been used to legitimize broader forms of social prejudice. In some cases this reveals underlying forms of prejudice within public health institutions, while in many cases this occurs as the media or general public appropriates and misuses the language of health authorities. In either situation public health authorities need to consider carefully the language that they use to describe health issues. Public health messages may inadvertently generate stigma through “content cues [that] encourage the activation of stereotypes, induce affective reactions (disgust, anger and fear), and associated action tendencies, which all foster the formation of stigma attitudes” [34], p463. These content cues can quickly take on a form of contagion in themselves if people share their fears with others and circulate messages about the perceived threats of the stigmatized group.

After learning from mistakes made in the 1980s, HIV/AIDS programmes have much to teach malaria elimination about the importance of language. In the United States for example, health authorities announced that homosexuals, heroin addicts, haemophiliacs and Haitians were the key risk groups and likely transmitters of HIV to the United States. This framing of people as high risk groups and threats to public health fuelled existing forms of racism and homophobia [36]. HIV/AIDS activists and scholars have succeeded to a great extent in reducing such fear and stigmatization over the years. This occurred in large part by avoiding the use of inflammatory language and insisting on a shift from identifying risk groups to identifying risk situations [37, 38]. Although the stigma surrounding HIV/AIDS continues and is embedded in many entrenched forms of social prejudice, this shift in language use helped to reframe the ways in which the public understands and responds to people living with HIV/AIDS and greatly decreased the ways that public health authorities were implicated in the stigma surrounding HIV/AIDS.

A more recent example of the potential for public health discourses to stigmatize groups lies in the controversy surrounding the naming of the H1N1 influenza pandemic in 2009. The virus was initially named Mexican flu, a move which was immediately rejected by Mexican health authorities who did not want Mexico to be seen as responsible for a pandemic [39]. Many then began to refer to the virus as swine flu, which incorrectly implied that the virus could be transmitted to humans by pigs. The WHO was cognizant of the political debates emerging around the naming of the flu and tried to insist the virus be named H1N1 [40]. The virus was nonetheless colloquially referred to as swine flu in the media and in much scientific coverage, fuelling debate within the Arab world [41]. Israeli officials said that the name was degrading to both Muslims and Jews, while another Israeli newspaper referred to the virus as “a symptom of the illness of Israeli politics” [39], p231. Contrary to the advice of the WHO and despite having no cases of H1N1, the Egyptian government ordered the culling of several hundred thousand pigs. This economically disadvantaged the Christian minority and added a public health justification to the ongoing marginalization of this minority group [41]. Although influenza is not a stigmatized disease in itself, the language surrounding swine flu easily entered media discourse and the popular imagination and became a discursive tool that further entrenched the marginalization of a minority group.

The language that is currently used within malaria elimination discourse has the potential to stigmatize vulnerable groups who are seen as responsible for ongoing transmission. Describing people as hotpops and reservoirs of infection establishes particular groups not as people to be protected from malaria and benefit from elimination, but to the contrary as sources of infection and threats to public health. As Williams and colleagues [14] point out, the elimination literature consistently describes displaced persons as a source of imported malaria, while little attention is given to the health needs of this group. While recognizing that accurate surveillance is important for achieving elimination, a strong focus on migrants as the primary source of ongoing malaria transmission and on poor border communities as ‘reservoirs of infection’ established a basis for a disproportionate blaming of malaria persistence on migrants and other vulnerable groups. As surveillance and mapping technologies advance, it is now possible to identify sources of infection down to a specific household or individual [42]. While this means more accurate surveillance, it also increases the risk that particular groups of people can be blamed for a future outbreak. While such surveillance technologies will be important for achieving elimination, these should be developed in ways that minimize possible adverse social impact. More than identifying preferable terminology, this will involve a broader shift in orientation from identifying risk groups as a problem to be addressed towards engaging with communities as partners in elimination.

### Malaria elimination can be achieved without stigmatization

While expressing concern about the potential for malaria elimination programmes to inadvertently generate stigma, the authors are certainly not arguing against malaria elimination but to the contrary believe that it is possible to pursue elimination without stigmatization. There are both ethical and pragmatic reasons why it is advantageous for elimination programmes to take measures to reduce the adverse social impact of elimination programmes. Stigma adds to the burden of disease by increasing personal suffering and adding to social inequalities [25, 26, 43, 44]. In some cases stigma leads people to avoid health services or even to reject public health programmes [30, 43]. Castro and Farmer argue that stigma arises within the same forms of structural violence that lead certain groups to be more vulnerable to disease, and as such there is a moral imperative to reduce stigma at the same time as reducing disease [44]. Weiss [26] argues that public health programmes should aim to “transform stigma into social support,” so that the observation of negative social impact becomes a point through which to develop better programmes.

In the first instance, this should involve avoiding the use of highly negative language such as ‘hotpops’ and ‘reservoirs of infection,’ that explicitly identifies particular groups as a cause of ongoing transmission. This is particularly urgent in situations where migrants are described as sources of imported malaria while their health needs remain marginal to discussion [14, 35]. Although cross-border movement is sometimes normalized at a local level [18, 45], discrimination against migrants is very common in many parts of the world and malaria elimination programmes should take care not to amplify these forms of discrimination. While recognizing that it is easier to identify problematic language than to choose better terminology to describe populations [46], this is essential to delivering more effective elimination programmes. Preferable terminology would be aimed not only at identifying populations that pose a risk to elimination, but at opening avenues to provide better services to people at risk of malaria and working more constructively with communities within elimination efforts. Note that this involves not just new terminology but a greater incorporation of community engagement strategies into elimination efforts.

In addition, the authors recommend that elimination programmes implement monitoring and evaluation of activities to assess the social impact of elimination programmes. While this commentary warns of the potential for elimination discourse to lead to social harm, it is only through evaluating programmes and carrying out social impact assessments that programmes can ascertain whether their activities in fact carry unintended social implications. As programmes become more targeted in local areas, strengthened community engagement may also add local knowledge to programmes and help programmes become more responsive to localized dynamics influencing malaria transmission. Since resources often become constrained in elimination settings, it will be necessary to continue advocating for sufficient resourcing to enable elimination efforts to continue and to reduce the risk that outbreaks may be blamed upon communities. Rather than risk profiling and targeting narrow demographic groups, strengthened community engagement efforts will help to ensure that programmes provide accurate information to communities about the value of pursuing malaria elimination. This will help to ensure that communities nearing elimination can respond to any future disease outbreaks in ways that decreases the risk that elimination programmes will contribute to social harm but rather help to strengthen elimination efforts.
